# TRPV4-Mediated Regulation of the Blood Brain Barrier Is Abolished During Inflammation

**DOI:** 10.3389/fcell.2020.00849

**Published:** 2020-08-27

**Authors:** Sina C. Rosenkranz, Artem Shaposhnykov, Oliver Schnapauff, Lisa Epping, Vanessa Vieira, Karsten Heidermann, Benjamin Schattling, Volodymyr Tsvilovskyy, Wolfgang Liedtke, Sven G. Meuth, Marc Freichel, Mathias Gelderblom, Manuel A. Friese

**Affiliations:** ^1^Institut für Neuroimmunologie und Multiple Sklerose, Universitätsklinikum Hamburg-Eppendorf, Hamburg, Germany; ^2^Klinik und Poliklinik für Neurologie, Universitätsklinikum Hamburg-Eppendorf, Hamburg, Germany; ^3^Klinik für Neurologie mit Institut für Translationale Neurologie, Universität Münster, Münster, Germany; ^4^Pharmakologisches Institut, Universität Heidelberg, Heidelberg, Germany; ^5^Departments of Neurology, Anesthesiology and Neurobiology, Duke University Medical Center, Durham, NC, United States

**Keywords:** TRPV4, transendothelial resistance, blood brain barrier, experimental autoimmune encephalomyelitis, stroke

## Abstract

Blood-brain barrier (BBB) dysfunction is critically involved in determining the extent of several central nervous systems (CNS) pathologies and here in particular neuroinflammatory conditions. Inhibiting BBB breakdown could reduce the level of vasogenic edema and the number of immune cells invading the CNS, thereby counteracting neuronal injury. Transient receptor potential (TRP) channels have an important role as environmental sensors and constitute attractive therapeutic targets that are involved in calcium homeostasis during pathologies of the CNS. Transient receptor potential vanilloid 4 (TRPV4) is a calcium permeable, non-selective cation channel highly expressed in endothelial cells. As it is involved in the regulation of the blood brain barrier permeability and consequently cerebral edema formation, we anticipated a regulatory role of TRPV4 in CNS inflammation and subsequent neuronal damage. Here, we detected an increase in transendothelial resistance in mouse brain microvascular endothelial cells (MbMECs) after treatment with a selective TRPV4 inhibitor. However, this effect was abolished after the addition of IFNγ and TNFα indicating that inflammatory conditions override TRPV4-mediated permeability. Accordingly, we did not observe a protection of *Trpv4*-deficient mice when compared to wildtype controls in a preclinical model of multiple sclerosis, experimental autoimmune encephalomyelitis (EAE), and no differences in infarct sizes following transient middle cerebral artery occlusion (tMCAO), the experimental stroke model, which leads to an acute postischemic inflammatory response. Furthermore, Evans Blue injections did not show differences in alterations of the blood brain barrier (BBB) permeability between genotypes in both animal models. Together, TRPV4 does not regulate brain microvascular endothelial permeability under inflammation.

## Introduction

The blood-brain barrier (BBB) is responsible for maintaining the separation of the central nervous system (CNS) from the blood and thereby controls the entry of neurotoxic metabolites, ions, pathogens and blood cells (Zhao et al., 2015). The cells of the BBB communicate with cells of the CNS but also with circulating immune cells allowing a tight adaption to maintain the highly regulated CNS internal milieu (Banks, 2016). The major component of the BBB are pericytes, astrocytes and endothelial cells that control the passage of molecules by a crosstalk between tight and adherens junctions (Obermeier et al., 2013; Keaney and Campbell, 2015; Tietz and Engelhardt, 2015).

Diminished BBB integrity results in increased vascular permeability and is essential for the development of cerebral edema (Sweeney et al., 2018). Similarly, a breakdown of the BBB facilitates the entry of immune cells into the CNS and is crucial for neuroinflammation (Man et al., 2007; Bennett et al., 2010; Ransohoff and Engelhardt, 2012). Proinflammatory cytokines evoke an upregulation of cell adhesion molecules on endothelial cells which can bind to integrins on leukocytes and thereby facilitate transendothelial leukocyte migration (Minagar and Alexander, 2003; Keaney and Campbell, 2015). Attenuating BBB permeability reduces the number of invading immune cells and the extent of a subsequent edema, which then ameliorates neuronal loss. Interventional studies in animal models of multiple sclerosis (Göbel et al., 2019; Viñuela-Berni et al., 2020) and stroke (Casas et al., 2017; Pan et al., 2020) where the BBB permeability was decreased as a result of treatment showed improved neurological outcomes. However, until now no clinically approved drug exists, highlighting the need to identify new effective targets.

A promising candidate which is known to regulate the integrity of several endo- and epithelial barriers, is the transient receptor potential vanilloid 4 (TRPV4), which is a calcium-permeable non-selective cation channel and a member of the transient receptor potential (TRP) superfamily of cation channels (Liedtke et al., 2000; Strotmann et al., 2000; Wissenbach et al., 2000). TRP channels play an important role as environmental sensors and constitute an attractive gene family that could be involved in CNS pathologies (Schattling et al., 2012; Gelderblom et al., 2014; Kanju and Liedtke, 2016). *Trpv4* is expressed widely including endothelial cells and astrocytes. TRPV4 is polymodally activated (Nilius et al., 2004; Moore et al., 2018) by multiple extracellular mechanic stimuli such as cellular shear, stretch and cell compression (Michalick and Kuebler, 2020), by osmotic changes (Liedtke et al., 2000; Liedtke, 2005), by thermal cues (Tominaga and Caterina, 2004), by UVB radiation (Moore et al., 2013), by endogenous bioactive lipids such as arachidonic acids metabolites (Watanabe et al., 2003) and others. However, the molecular mechanisms of TRPV4 activation could differ from cell-type to cell-type and are overall not completely understood. In endothelial cells, TRPV4 functions as mechanoreceptor in response to shear stress (Matthews et al., 2010). Upon TRPV4 activation calcium fluxes into the cell and activates calcium-activated potassium channels. This can result in increased barrier permeability (Morty and Kuebler, 2014). It was shown that TRPV4 inhibition prevents and resolves pulmonary edema (Hamanaka et al., 2010; Thorneloe et al., 2012; Michalick et al., 2017), reduces the infarct size in myocardial ischemia (Dong et al., 2017), decreases the vascular endothelial permeability in murine colitis (Matsumoto et al., 2018) and diabetic retinal injury (Arredondo Zamarripa et al., 2017; Ríos et al., 2019). Furthermore, TRPV4 also regulates the integrity of the blood-cerebrospinal fluid barrier (Narita et al., 2015) and TRPV4 inhibition reduced BBB disruption and consecutive edema in a mouse model of intracerebral hemorrhage and thereby ameliorated neurological symptoms (Zhao et al., 2018). These observations imply an important role of TRPV4 in the BBB.

Therefore, modifications of TRPV4 could constitute an attractive therapeutic target in diseases with neuroinflammatory pathways that rely on temporarily impaired BBB integrity for influx of disease-enhancing immune cells. Here we analyzed whether TRPV4 inhibition has an impact on transendothelial resistance under homeostatic and inflammatory conditions and whether deletion of *Trpv4* affects the clinical outcome in mouse models of multiple sclerosis and stroke. We show that inflammation overrules the homeostatic TRPV4-mediated regulation of the BBB, and that *Trpv4* deficiency does not modify the outcome in these disease models.

## Materials and Methods

### Mice

All mice [C57BL/6J wild-type, The Jackson Laboratory; *Trpv4*^–/–^ mice on a C57BL/6J genetic background (Liedtke and Friedman, 2003)] were kept under specific pathogen-free conditions in the central animal facility of the Universitätsklinikum Hamburg-Eppendorf (UKE). Animals were housed in a facility with 55–65% humidity at 24 ± 2°C with a 12-h light/dark cycle and had free access to food and water.

### EAE Induction

Mice were anaesthetized with isoflurane 1–2% v/v oxygen and immunized subcutaneously with 200 μg myelin oligodendrocyte glycoprotein 35–55 (MOG_35–55_) peptide (peptides & elephants) in complete Freund’s adjuvant (BD) containing 4 mg ml^–1^ *Mycobacterium tuberculosis* (BD). 200 ng pertussis toxin (Merck) was injected intravenously on the day of immunization and 48 h later. Animals were scored daily for clinical signs by the following system: 0, no clinical deficits; 1, tail weakness; 2, hind limb paresis; 3, partial hind limb paralysis; 3.5, full hind limb paralysis; 4, full hind limb paralysis and forelimb paresis; 5, premorbid or dead. Animals reaching a clinical score ≥ 4 were sacrificed according to the regulations of the Animal Welfare Act. The experimenters were blinded to the genotype until the end of the experiment, including data analysis. Sex- and age-matched adult animals (8–12 weeks of age) were used in all experiments. For *Trpv4*^–/–^ mice and WT controls, three independent EAE experiments were conducted. The data were pooled for final analysis. For analysis of the disease course and weight we only included animals which received a score ≥ 1 until day 20 and survived until day 30. For analysis of onset we included all mice which received a score ≥ 1.

### Determination of Blood Brain Barrier Permeability in EAE

BBB integrity was measured at the peak (day 13 after immunization) of EAE (*n* = 5 per genotype). Mice were intravenously injected with 200 μl of 2% Evans Blue (Millipore Sigma) dissolved in 0.1 M phosphate buffer saline (PBS). 2 h later mice were anesthetized intraperitoneally with 100 μl solution (10 mg ml^–1^ esketamine hydrochloride (Pfizer), 1.6 mg ml^–1^ xylazine hydrochloride (Bayer) dissolved in water) per 10 g of body weight and perfused with PBS. Brain, spinal cord and kidney were resected, dried for 24 h at 50°C and placed into formamide (Sigma) the volume of which was adjusted to the tissue weight. For the complete Evans Blue extraction, samples were incubated 24 h at 55°C. Amount of dye was determined by colorimetric analysis as absorbance coefficient at 610 nm. The data for brains and spinal cords were normalized to the kidneys values (Radu and Chernoff, 2013).

### Mouse Tissue Preparation and Histopathology of EAE Mice

Mice were anesthetized intraperitoneally with 100 μl solution (10 mg ml^–1^ esketamine hydrochloride (Pfizer), 1.6 mg ml^–1^ xylazine hydrochloride (Bayer) dissolved in water) per 10 g of body weight. Afterward mice were perfused with 4% paraformaldehyde (PFA) and cervical spinal cord was resected, dehydrated and cast in paraffin. CD3 staining (rabbit IgG, Abcam ab16669) was visualized by the avidin-biotin technique with 3,3-diaminobenzidine according to standard procedures of the UKE Mouse Pathology Facility. The slides were analyzed using a NanoZoomer 2.0-RS digital slide scanner and NDP.view2 software (Hamamatsu). Quantification of infiltrating CD3 cells was done with ImageJ software (NIH), using the same settings across experimental groups.

### *In vivo* Stroke Model

Mice were anesthetized using isoflurane 1–2% v/v oxygen and we injected buprenorphine 0.03 mg/kg body weight intraperitoneally (i.p.) every 12 h for 24 h as analgesia. We conducted transient middle cerebral artery occlusion (tMCAO) for 45 min using the intraluminal filament method (6-0 nylon, Docoll) as described before (Gelderblom et al., 2014). We monitored mice for heart rate, respiratory rate, oxygen saturation, rectal body temperature, and cerebral blood flow by using transcranial temporal laser Doppler technique. Only mice with a sufficient decrease in the ipsilateral laser Doppler flow (below 20 % when compared to the contralateral site) were included in the study, to ensure an appropriate occlusion of the middle cerebral artery. Animals were scored immediately after reawakening and daily for clinical signs by the following system (Bederson Score): 0, no deficit; 1, preferential turning; 2, circling; 3, longitudinal rolling; 4, no movement; 5, death. For *Trpv4*^–/–^ mice and WT controls, two independent stroke experiments were conducted. The data were pooled for final analysis. Mortality did not differ between the groups. To reduce the variability of our outcome parameters caused by sex-differences only male mice (12–16 weeks of age) were used throughout the study.

### Analysis of Infarct Size by TTC Staining

Only mice with a Bederson score greater or equal than one after reawakening and a sufficient occlusion of the middle cerebral artery during MCAO as measured by laser Doppler technique were included in stroke size analysis. Mice were sacrificed 3 days after reperfusion using isoflurane and decapitation. Brains were harvested and cut into 1 mm slices (Braintree Scientific, 1 mm) followed by vital staining using 2% (wt/vol) 2,3,5-triphenyl-2-hydroxy-tetrazolium chloride (TTC) in phosphate buffer. We determined infarct volumes blinded for genotype by using NIH ImageJ software.

### Determination of Blood Brain Barrier Permeability in Stroke

BBB integrity following stroke was assessed in mice (*n* = 6 per experimental group) that were intraperitoneally injected with 200 μl of 2% Evans Blue (Millipore Sigma, Cat. No. E2129) dissolved in 0.1 M phosphate buffer saline (PBS) directly after tMCAO. 24 h later mice were processed as described above. We additionally analyzed Evans Blue staining after 72 h of tMCAO.

### Primary Endothelial Culture and Transendothelial Electrical Resistance (TEER)

Mouse brain microvascular endothelial cells (MbMECs) were isolated as previously described (Weidenfeller et al., 2005; Ruck et al., 2014) and incubated in a humidified incubator with 5% CO_2_ at 37°C. Fresh, puromycin free MbMEC medium was added 4 days after isolation. Two days later, when cells reached confluence, they were harvested by trypsinization and seeded for subsequent TEER experiments onto pre-coated transwell inserts (pore size 0.4 μm; Corning) at 2 × 10^4^ cells per insert. TEER measurements were performed and analyzed using the cellZscope 24-cell module and cellZscope v2.2.2 software, respectively (nanoAnalytics GmbH) as described before (Kuzmanov et al., 2016). Automated TEER and cell layer capacitance (Ccl) measurements were performed every hour for 3 to 4 days until the MbMEC monolayer reached full confluence, as determined by stable Ccl below 1 μF/cm^2^ and TEER at its maximum plateau for at least 6 h. At that time point, MbMECs were either kept naïve or inflammation was induced by addition of 50 U/ml IFNγ and 50 U/ml TNFα. Additionally, either vehicle or GSK2193874 (Sigma), a TRPV4-specific inhibitor, which has been shown to inhibit TRPV4-dependent calcium influx in endothelial cells and exerts potent *in vivo* activity (Thorneloe et al., 2012; Cheung et al., 2017), were applied and TEER measurements were resumed for another 24 h.

### Expression Analysis

For analysis of *Trpv4* mRNA expression on MbMECs, total RNA was extracted from naïve MbMECs or MbMECs inflamed for 24 h with 50 U/ml IFNγ and 50 U/ml TNFα by using a Quick RNA Micro Prep Kit (Zymo Research); cDNA was synthesized from 300 ng of total RNA by using a Maxima First Strand cDNA Synthesis Kit (ThermoFisher Scientific), all performed according to the manufacturers’ instructions. For quantitative RT-PCR (RT-qPCR), a mouse *Trpv4* TaqMan Gene Expression Assay (Mm00499025_m1) with 18S rRNA as endogenous control was used. RT-qPCR was performed by using the StepOnePlus System (Applied Biosystems). Data were analyzed using the ΔΔCT method followed by relative quantification (2^–ΔΔCT^).

### Study Approval

All animal care and experimental procedures were performed according to institutional guidelines and conformed to requirements of the German Animal Welfare Act. All animal experiments were approved by the local ethics committee (Behörde für Soziales, Familie, Gesundheit und Verbraucherschutz in Hamburg; G22/13 and 59/17. We conducted all procedures in accordance with the ARRIVE guidelines (Kilkenny et al., 2010).

### Statistics

Experimental data were analyzed using Prism 8 software (GraphPad) and are presented as mean values ± SEM. Statistical analyses were performed using the appropriate test indicated in the figure legends. D’Agostino and Pearson test was used to analyze normality. Unless stated otherwise, differences between two experimental groups were determined by unpaired, two-tailed Mann-Whitney or Students *t*-test. Significant results are indicated by asterisks: ^∗^*P* < 0.05.

## Results

### TRPV4 Inhibition Increases Transendothelial Resistance

**FIGURE 1:**
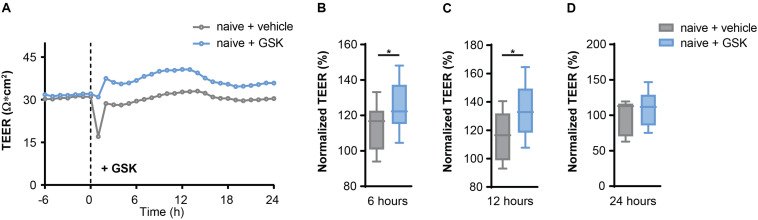
TRPV4 inhibition increases transendothelial electrical resistance (TEER) in mouse brain microvascular endothelial cells (MbMECs) under homeostatic conditions. **(A)** TEER of MbMECs treated with 5 nM of GSK2193874 in comparison to vehicle-treated MbMECs. One representative experiment out of 12 is shown. **(B–D)** Quantification of TEER of MbMECs treated with 5 nM of GSK2193874 (*n* = 12) in comparison to vehicle treated MbMECs (*n* = 12) at 6, 12, and 24 h. TEER was normalized to the time-point of GSK treatment (0 h). Data are presented as box plots. Statistical analysis was performed by two-tailed Student’s *t*-test. *P < 0.05.

First, we validated a regulative role of TRPV4 for BBB permeability. We measured the transendothelial electrical resistance (TEER) of mouse brain microvascular endothelial cells (MbMECs) in the absence and presence of the specific pharmacological TRPV4 inhibitor GSK2193874, which has been previously shown to have potent efficiency in ameliorating lung edema (Thorneloe et al., 2012). Six and 12 h after addition of GSK2193874 we detected a significant increase of the TEER, which was not anymore detectable after 24 h (Figures 1A–D). Thus, TRPV4 inhibition by GSK2193874 induces an increased endothelial cell barrier integrity.

### Inflammation Overrides TRPV4-Mediated Permeability of the Blood Brain Barrier

**FIGURE 2:**
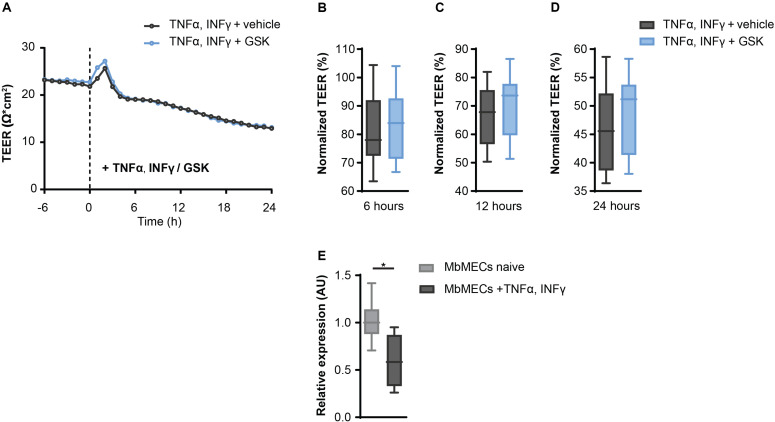
Inflammatory conditions abolish the effect of TRPV4 inhibition on transendothelial electrical resistance (TEER) in mouse brain microvascular endothelial cells (MbMECs). **(A)** TEER of MbMECs treated with 5 nM of GSK2193874 in comparison to vehicle-treated MbMECs, both were exposed to 50 U/ml TNFα and INFγ. One representative experiment out of 14 is shown. **(B–D)** Quantification of TEER of MbMECs treated with 5 nM of GSK2193874 (*n* = 14) in comparison to vehicle-treated MbMECs (*n* = 14) at 6, 12, and 24 h, both were exposed to 50 U/ml TNFα and INFγ. TEER was normalized to the time-point of GSK treatment (0 h). **(E)** Relative *Trpv4* mRNA expression assessed by qPCR in MbMECs exposed to 50 U/ml TNFα and INFγ (*n* = 4) in comparison to homeostatic conditions (*n* = 8). Data are presented as box plots. Statistical analysis was performed by two-tailed Student’s *t*-test **(A)** and **(B–D)** or two-tailed Mann Whitney test **(E)**. *P < 0.05.

Since TRPV4 inhibition leads to an increased resistance in MbMECs, we next investigated whether this could be confirmed under inflammatory conditions. We analyzed TRPV4 functionality after the exposure of tumor necrosis factor-α (TNFα) and interferon-γ (INFγ) that are key cytokines during neuroinflammation (Becher et al., 2017). Notably, in the presence of TNFα and INFγ the observed effect of TEER increase by inhibiting TRPV4 activity under homeostatic conditions was abolished (Figures 2A–D).

As changes in cytokine-mediated *Trpv4* expression could be an explanation for the abolished regulation, we next analyzed *Trpv4* expression in MbMECs under the different conditions. Indeed, we observed a significant reduction of *Trpv4* mRNA in MbMECs after exposure to TNFα and INFγ in comparison to homeostatic conditions (Figure 2E). We concluded that inflammatory cytokines induce a TRPV4 loss-of-function in MbMECs by attenuating the gene expression of *Trpv4.*

### Blood Brain Barrier Permeability in EAE Is Not Altered by TRPV4

**FIGURE 3:**
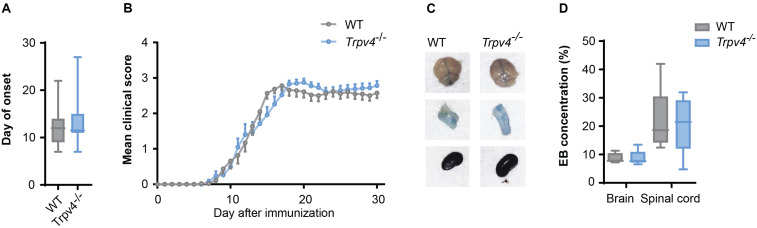
No difference between *Trpv4*^–/–^ mice and WT controls on the disease course and BBB permeability in EAE. **(A)** Day of onset of WT (*n* = 33) and *Trpv4*^–/–^ mice (*n* = 32) during the course of EAE. **(B)** Clinical scores of WT (*n* = 27) and *Trpv4*^–/–^ mice (*n* = 23) undergoing EAE. **(C)** Evans Blue (EB) quantification in brain and spinal cord of WT (*n* = 5) and *Trpv4*^–/–^ (*n* = 5) mice at day 13 after EAE immunization. Evans Blue concentration was normalized to the concentration of the right kidney of the corresponding animal. **(D)** Representative images of brain, spinal cord and kidney of WT- and *Trpv4*^–/–^-EAE mice 2 h after Evans Blue injection. Data in **(A,C)** are presented as box plots, in **(B)** as mean values ± s.e.m. Statistical analysis was performed by two-tailed Student’s *t*-test in **(A)** by two-tailed Mann Whitney test in **(B,C)**.

As TRPV4 inhibition leads to an increased resistance of endothelial cells, which is abrogated under inflammatory conditions, we next investigated whether TRPV4-mediated BBB regulation is indeed overruled in EAE. For this, we induced EAE in *Trpv4*-deficient (*Trpv4^–/–^)* mice and compared their disease course to wild-type (WT) controls. We did not see any differences in hallmark clinical phenotypes such as disease onset (Figure 3A), disease disability score (Figure 3B) or body weight changes (Supplementary Figure 1A).

Additionally, we assessed the BBB permeability by analyzing Evans Blue staining at the peak of disease. As this form of EAE mainly affects the spinal cord we observed higher levels of Evans Blue in the spinal cord than the brain at day 13 after immunization, without detecting a significant difference in the amount of Evans Blue in *Trpv4*^–/–^ mice in comparison to WT mice (Figures 3C,D). Moreover, we could not detect any differences in the numbers of infiltrating T cells (Supplementary Figures 1B,C).

### Blood Brain Barrier Permeability in tMCAO Is Not Altered by TRPV4

Given the absence of a phenotype and a difference in BBB permeability in *Trpv4*^–/–^ mice subjected to EAE, we decided to investigate the effects on experimental stroke, another model with significant impact of BBB disruption on clinical outcome but with more mildly and acute inflammation.

**FIGURE 4:**
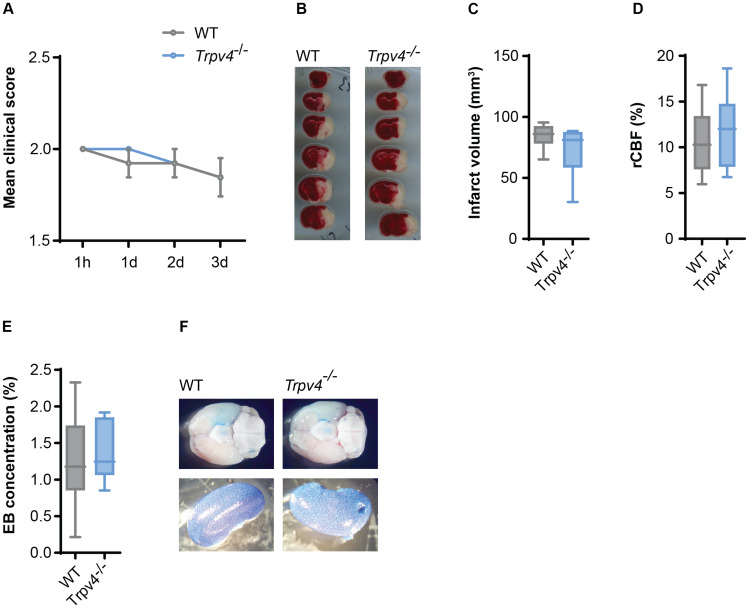
No difference between *Trpv4*^–/–^ mice and WT controls on disease course and BBB permeability in tMCAO. **(A)** Clinical scores of WT (*n* = 13) and *Trpv4*^–/–^ mice (*n* = 13) after tMCAO; h = hours, d = days. **(B)** Representative TTC staining of WT and *Trpv4*^–/–^brains 3 days after tMCAO. **(C)** Quantification of infarct volume of WT (*n* = 8) and *Trpv4*^–/–^ (*n* = 9) 3 days after tMCAO. **(D)** Regional cerebral blood flow (rCBF) of WT (*n* = 13) and *Trpv4*^–/–^ mice (*n* = 13) was measured during occlusion by laser Doppler and normalized to contralateral hemispheres. **(E)** Evans Blue (EB) quantification in brains of WT (*n* = 6) and *Trpv4*^–/–^ (*n* = 6) mice 24 h after tMCAO. Evans Blue concentration was normalized to the concentration of the right kidney of the corresponding animal. **(F)** Representative images of brains and kidneys of WT and *Trpv4*^–/–^ mice 24 h after tMCAO and Evans Blue injection. Data in **(A)** are presented as mean values ± s.e.m, in **(C–E)** as box plots. Statistical analysis was performed by two-tailed Mann Whitney test for (A) and two-tailed Student’s *t*-test for **(C–E)**.

Therefore, we induced cerebral ischemia by tMCAO in *Trpv4*^–/–^ and WT control mice and assessed their infarct sizes and neurological scores. After three days we were not able to detect any differences in the disease score between the two groups (Figure 4A) or in the body weight (Supplementary Figure 2A). There was also no difference in the volume of infarcted tissue (Figures 4B,C). Notably, both genotypes showed the same regional cerebral blood flow in the tMCAO model assessed by laser Doppler (Figure 4D) and were not different in physiological parameters (Supplementary Figures 2B–E).

Moreover, Evans Blue staining of the brain was assessed 24 h (Figures 4E,F) and 72 h (data not shown) after tMCAO, revealing lack of any differences between the two groups.

## Discussion

Here we show that TRPV4 inhibition increases endothelial resistance under homeostatic conditions, however, this effect was lost during inflammatory conditions. TRPV4 activation led to a disintegration of tight junctions (Narita et al., 2015) and TRPV4 inhibition decreases the vascular endothelial permeability and thereby counteracts pulmonary edema induced by heart failure (Thorneloe et al., 2012). Our data support these previously established concepts, as we detected a higher TEER in MbMECs after inhibiting TRPV4. Of note, inflammatory cytokines abolished this effect and led to a downregulation of *Trpv4* in MbMECs. Consistently, previous data showed that proinflammatory cytokines (e.g., TNF-α and IL-1β) had inhibitory effects on TRPV4-stimulated transepithelial ion flux and permeability changes in the choroid plexus, whereas anti-inflammatory cytokines (e.g., IL-10 and IL-4) showed no effect (Simpson et al., 2019). However, in this study the cytokines reduced only the functional but not the transcriptional regulation of TRPV4. Thus, it is likely a contribution of transcriptional regulation, alteration of TRPV4 channel function by trafficking and/or post-translational modification, and other complex regulation. Hence, further studies exploring the impact of inflammation on *Trpv4* expression and TRPV4 ion channel function in endothelial cells and their respective barrier are required.

In our study, we were not able to detect any differences in the *Trvp4-*deficient mice in EAE and tMCAO. Immune cell infiltration, microglia activation and increased level of proinflammatory cytokine release into the CNS accounts for the pathogenesis of both disease models (Lopes Pinheiro et al., 2016) but in comparison to EAE with abundant immune cell infiltration into the spinal cord, tMCAO is accompanied by less robust immune cell invasion of the cortex. We could not detect any difference of BBB permeability in *Trvp4-*deficient in comparison to WT control mice in both models.

We conclude that the inflammation overrides the possible protective effects of TRPV4. In line with our data, TRPV4 inhibition showed no protective effect in TNF-α induced sepsis, whereas it does in LPS induced sepsis (Dalsgaard et al., 2016). Therefore, it needs to be further specified which mechanisms and conditions lead to the abolishment of TRPV4 induced regulation of BBB permeability during inflammation.

Our lack of an *in vivo* phenotype might be rooted in several factors so that we are not claiming a decisively confirmed lack of a role of *Trpv4* in the examined animal models. *Trpv4* is also expressed on the other side of the endothelial side of the BBB, in astrocytes, in particular in astrocytic endfeet abutting the capillary (Shibasaki et al., 2007; Benfenati et al., 2011; Dunn et al., 2013; Filosa et al., 2013). Absence of astrocytic TRPV4 could alter the astrocytic contribution to the BBB. In that case the net result of absence of TRPV4 in endothelial cells and astrocytes could be lack of a phenotype in *Trpv4*-deficient mice because of opposite effects. In addition, there are other relevant cell migratory types such as macrophages that also express *Trpv4* and might contribute to the phenotype in various ways (Hamanaka et al., 2010; Scheraga et al., 2016; Michalick and Kuebler, 2020). Importantly, *Trpv4*-deficient mice have a genetically encoded absence of *Trpv4* in all cells at all developmental stages. For critical structures and function such as the BBB, this means compensatory gene expression to back up TRPV4 function might not be unlikely, plus cells that normally express *Trpv4* and have regulated expression of *Trpv4* under stress/injury can have a very different response, which is not directly rooted in absence of TRPV4, but rather in developmental compensation of gene expression. This uncertainty could be further addressed by studies in mice with lineage-specific gene targeting so that the deletion of *Trpv4* can be induced (Moore et al., 2013). Such dedicated studies can be combined with acute application of selective inhibitor molecules with systemic and compartmentalized application.

Beyond *Trpv4* expression in endothelial cells and astrocytes of the BBB, the channel appears to function in multiple roles and multiple lineages of the CNS (Lee and Choe, 2014; Kanju and Liedtke, 2016). For example, in a cuprizone-induced mouse model of demyelination a TRPV4 antagonist, RN-1734, alleviated demyelination and inhibited glial activation (Liu et al., 2018). Intracerebroventricular injection of HC-067047, a specific TRPV4 antagonist, reduced brain infarction 24 h after tMCAO (Jie et al., 2016). It appears therefore puzzling that TRPV4 activation using the selective activator 4α-PDD, induced angiogenesis and neurogenesis and thereby contributed to functional recovery from ischemic stroke in mice (Chen et al., 2017). These could be off-target effects of the different “specific” chemicals, but perhaps could highlight different signaling functions of TRPV4 in different lineages at different phases of ischemic injury of the CNS, characterized by different stages of BBB injury.

In summary, we demonstrate that TRPV4 inhibition leads to an increase of transendothelial resistance under homeostatic conditions, while this effect could not be observed under inflammatory conditions. In our *in vivo* models of EAE and acute cerebral ischemia, *Trpv4*-deficient mice were devoid of a phenotype, especially regarding BBB injury. These results build the foundation for a future quest for the role of TRPV4 in the BBB with focus on relevant constituting and TRPV4-expressing cells such as endothelial cells. However, our data do not support therapeutic TRPV4 channel inhibition in patients with multiple sclerosis or ischemic stroke.

## Data Availability Statement

All datasets generated for this study are included in the article/Supplementary Material.

## Ethics Statement

The animal study was reviewed and approved by the Behörde Für Soziales, Familie, Gesundheit und Verbraucherschutz in Hamburg; G22/13 and 59/17.

## Author Contributions

SR and AS conducted and designed the experiments, analyzed the data, and wrote the manuscript. OS, VV, LE, and KH conducted the experiments and analyzed the data. BS, SM, and MF designed the experiments. WL interpreted the data and wrote the manuscript. MG and MAF designed the experiments, conceived, supervised, and funded the study, and wrote the manuscript. All authors contributed to the article and approved the submitted version.

## Conflict of Interest

The authors declare that the research was conducted in the absence of any commercial or financial relationships that could be construed as a potential conflict of interest.
