# Supplementary material for: TRPV4-Mediated Regulation of the Blood Brain Barrier Is Abolished During Inflammation
Source: Front Cell Dev Biol. 2020 Aug 27;8:849. doi: 10.3389/fcell.2020.00849 (PMC7481434; doi:10.3389/fcell.2020.00849)
Supplement: Supplementary file 1 [file Image_1.pdf]

Supplementary Material

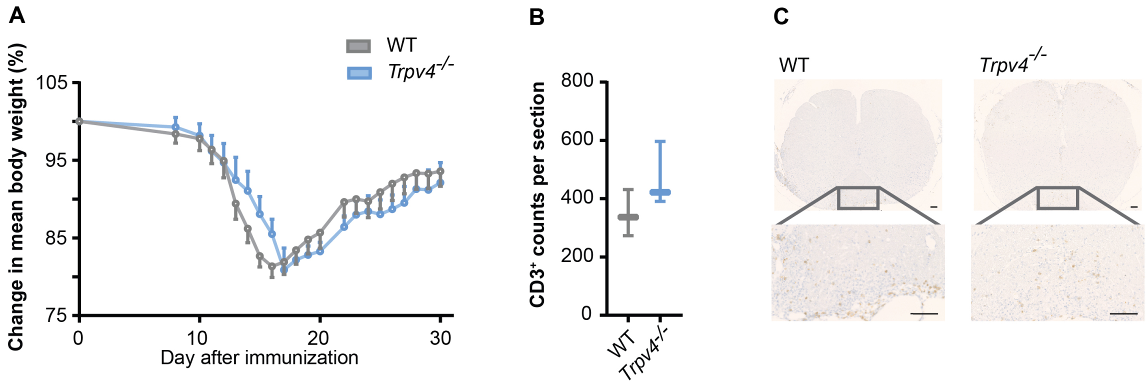

Supplementary Figure 1. TRPV4 activity does not alter body weight or immune cell infiltration during EAE.
(**A**) Weight loss normalized to baseline weight of WT (n = 27) and *Trpv4*$^{-/-}$ mice (n = 23) undergoing EAE.
(**B**) Quantification of histopathological stainings of T cells (CD3) in cervical spinal cord sections of WT (n = 3) and *Trpv4*$^{-/-}$ (n = 3) mice at day 15 post immunization with quantifications.
(**C**) Representative images of histopathological stainings of T cells (CD3) in cervical spinal cord sections of WT and *Trpv4*$^{-/-}$-EAE mice. Scale bar 100µm.
Data are presented as box plots. Statistical analyses were performed by two-tailed Student's t-test for (A) and two-tailed Mann Whitney test for (B).

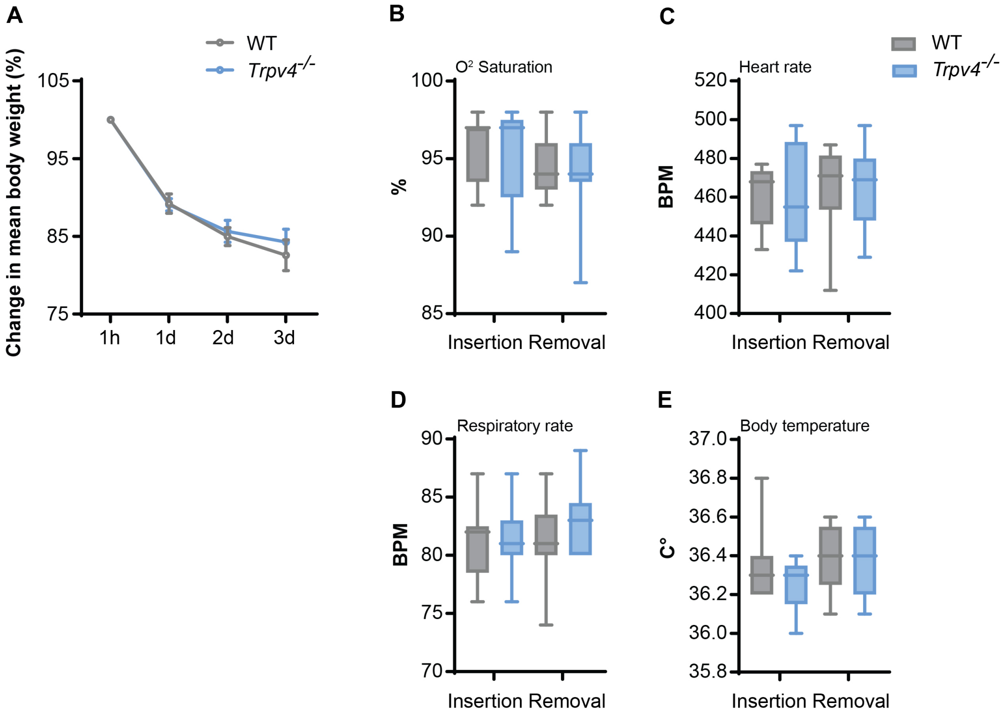

Supplementary Figure 2. TRPV4 activity does not alter body weight and physiological parameters in tMCAO.
(**A**) Weight loss normalized to baseline weight of WT (n = 13) and *Trpv4*$^{-/-}$ mice (n = 13) after tMCAO; h = hours, d = days.
(**B**) O2 Saturation before and after occlusion in WT (n =13) and *Trpv4*$^{-/-}$ mice (n = 13).
(**C**) Heart rate before and after occlusion in WT (n =13) and *Trpv4*$^{-/-}$ mice (n = 13).
(**D**) Respiratory rate before and after occlusion in WT (n =13) and *Trpv4*$^{-/-}$ mice (n = 13).
(**E**) Body temperature before and after occlusion in WT (n =13) and *Trpv4*$^{-/-}$ mice (n = 13).
Data are presented as box plots. Statistical analyses were performed by two-tailed Student's t-test.
